# Clustering of Lifestyle Behaviors and Their Association With Risk of Metabolic Syndrome Among Adults in Taiwan: Nationwide Cross-Sectional Study

**DOI:** 10.2196/73114

**Published:** 2025-09-18

**Authors:** Ya-Hui Chang, Chung-Yi Li, Hon-Ping Ma, Chien-Yuan Wu, Yann-Yuh Jou, Chiachi Bonnie Lee

**Affiliations:** 1 Graduate Institute of Injury Prevention and Control, College of Public Health, Taipei Medical University, Taipei, Taiwan; 2 Emergency Department, Shuang Ho Hospital, Taipei Medical University, New Taipei City, Taiwan; 3 Department of Public Health, College of Medicine, National Cheng Kung University, Tainan, Taiwan; 4 Department of Public Health, College of Public Health, China Medical University, Taichung, Taiwan; 5 Department of Healthcare Administration, College of Medical and Health Science, Asia University, Taichung, Taiwan; 6 Department of Emergency, School of Medicine, College of Medicine, Taipei Medical University, Taipei, Taiwan; 7 Aging and Chronic Disease Control Division, Health Promotion Administration, Ministry of Health and Welfare, Taipei, Taiwan; 8 Department of Health Services Administration, College of Public Health, China Medical University, 100 Section 1, Jingmao Road, Beitun District, Taichung, 406040, Taiwan

**Keywords:** health promotion, latent class analysis, lifestyle patterns, metabolic syndrome, physical activity

## Abstract

**Background:**

Metabolic syndrome (MetS) is a multifaceted health condition influenced by physiological and lifestyle factors, leading to increased risks of cardiovascular disease and other chronic health issues. Lifestyle behaviors often manifest in various clustering patterns, and evidence of their impact on MetS remains limited.

**Objective:**

This study explores the relationship of latent classes of lifestyle behaviors with the risk of MetS and its components.

**Methods:**

This cross-sectional study used data from Taiwan’s 2020‐2022 Adult Preventive Health Services Database, which was linked to 2020‐2022 National Health Insurance claim data. The study included 241,156 adults aged 40 years and older who participated in adult preventive health services between 2020 and 2022. Lifestyle behaviors were assessed through smoking, alcohol consumption, betel quid chewing, and physical activities. Latent class analysis was used to identify lifestyle behavior patterns, while binary logistic regression examined the association of these patterns with MetS risk and its components.

**Results:**

The latent class analysis identified 5 distinct lifestyle behavior patterns, with an overall MetS prevalence of 35.72% (86,143/241,156). Compared to the “healthy lifestyle” group (27,465/241,156, 11.39% prevalence), the “insufficiently physically active (IPA)” group (182,101/241,156, 75.51%, adjusted odds ratio [aOR] 1.41, 95% CI 1.37‐1.45; *P*<.001), the “occasional drinking but physically active” group (18,244/241,156, 7.57%, aOR 1.27, 95% CI 1.21‐1.32; *P*<.001), the “occasional drinking and regular smoking with IPA” group (9539/241,156, 3.96%, aOR 2.38, 95% CI 2.26‐2.50; *P*<.001), and the “unhealthy in all behaviors” group (3807/241,156, 1.58%, aOR 2.38, 95% CI 2.22‐2.55; *P*<.001) showed significantly higher odds of developing MetS. Compared to the “healthy lifestyle” group, all other lifestyle patterns were also associated with significantly higher odds of central obesity (*P*<.001), elevated blood pressure (*P*<.001), elevated fasting blood glucose (*P*<.001), elevated fasting triglycerides (*P*<.001), and reduced high-density lipoprotein cholesterol (*P*<.001), with the most potent effects observed in the “occasional drinking and regular smoking with IPA” group and the “unhealthy in all behaviors” group. An exception was noted for the “occasional drinking but physically active” group, which showed a significantly lower likelihood of reduced high-density lipoprotein cholesterol (aOR 0.90, 95% CI 0.85‐0.94; *P*<.001).

**Conclusions:**

Engaging in sufficient physical activity and adopting multibehavior interventions tailored to specific lifestyle patterns are crucial for effectively preventing MetS in adults.

## Introduction

Metabolic syndrome (MetS) is a multifactorial disorder marked by a cluster of interrelated metabolic abnormalities, including central obesity, insulin resistance, and elevated blood pressure [1]. These conditions synergistically elevate the risk of type 2 diabetes, cardiovascular disease, stroke, and cognitive impairment, making MetS a critical global public health issue [2]. MetS has become a significant public health concern in Taiwan, with approximately 34.2% of adults aged over 50 years affected by this condition [3]. This high prevalence underscores the urgent need for effective prevention and intervention strategies.

The etiology of MetS extends beyond biological predispositions, involving a complex interplay of lifestyle and environmental exposure. In East and Southeast Asia, including Taiwan, behavioral risk factors such as tobacco use, alcohol consumption, physical inactivity, unhealthy dietary habits, and betel quid chewing are especially prevalent and contribute substantially to cardiometabolic disease burden [4,5]. In Taiwan, the prevalence of smoking among adults aged 18 years and over was reported at 12.8% in 2024, with higher rates observed among men [6]. Betel quid chewing remains a notable concern, particularly among men, with prevalence rates ranging from 1.6% to 15.4% across various regions [7]. Betel quid chewing has been linked to oxidative stress, inflammation, and insulin resistance, yet it remains under-investigated in MetS research [8].

Emerging research has shifted from evaluating isolated lifestyle factors to examining how unhealthy behaviors cluster within individuals [9,10]. This clustering effect is known to amplify health risks beyond the additive effects of single behaviors. Some studies have applied healthy lifestyle indices or scores to quantify these risks, such as composite metrics that often dichotomize behaviors (eg, active vs inactive), potentially obscuring nuanced behavior patterns and underestimating outcomes variability [10-12].

Latent class analysis (LCA) offers a more sophisticated approach to uncover underlying subgroups of individuals who share similar patterns of lifestyle behaviors [13]. This person-centered methodology can reveal heterogeneity in health risk profiles that traditional methods overlook. However, few population-based studies have applied LCA to investigate lifestyle behavior patterns, including culturally specific practices, like betel quid chewing, related to MetS and its components among Taiwanese adults. Therefore, this study aimed to identify lifestyle behavior patterns using LCA and assess their associations with MetS and its components in a population-based study of Taiwanese adults, with particular attention to the role of betel quid chewing.

## Methods

### Study Design

This nationwide cross-sectional study analyzed data from the Adult Preventive Health Services Database (APHSD), which was linked to National Health Insurance (NHI) claims data from 2020 to 2022. The study adhered to the Strengthening the Reporting of Observational Studies in Epidemiology guidelines for cross-sectional research (see Checklist 1).

### Data Source and Study Population

This study analyzed data from the APHSD, obtained from the Health Promotion Administration, Ministry of Health and Welfare (MOHW), Taiwan, which were linked to NHI claims data from 2020 to 2022 managed by the Health and Welfare Data Science Center (HWDC), MOHW. The service offers free health checkups for residents aged 40 years and older and individuals with a history of polio aged 35 years and older at clinics and hospitals in Taiwan. The study included individuals aged 40 years and older who accessed adult preventive health services during this period. Only the most recent record was retained for those with multiple visits, resulting in an initial dataset of 4,502,104 individuals. After excluding individuals with polio (*International Classification of Diseases, Tenth Revision, Clinical Modification* [*ICD-10-CM*]: A80.0-A80.2, A80.30-A80.39), individuals with missing data, or those identified as outliers (483,051 individuals), a final pool of 4,019,053 participants was established. A stratified random sampling method was applied, selecting 6% of that final participant pool based on gender and 5-year age intervals, leading to a final sample size of 241,156 individuals. As shown in Table S1 in Multimedia Appendix 1, there were no significant differences between the sample population and the overall population.

### Lifestyle Variables

This study included four lifestyle behaviors for this analysis: physical activity, cigarette smoking, alcohol drinking, and betel quid chewing status in the past 6 months. Cigarette smoking status was collected by answering the question, “In the past six months, what is your smoking status?” (Answer options: no smoking, smoking only during social occasions, smoking one or less pack per day, and smoking more than one pack per day). Alcohol consumption was measured by the question, “In the past six months, what is your alcohol drinking status?” (Answer options: no drinking, drinking only during social occasions, and regular drinking). Participants reported their betel quid chewing status through the question, “In the past six months, what is your betel quid chewing status?” (Answer options: no chewing, chewing only during social occasions, and regular chewing). Physical activity was measured with the following question: “In the last two weeks, have you done any physical activity for more than 150 minutes per week?” (Answer options: no; yes, but less than 150 minutes per week; yes, and more than 150 minutes per week). We categorized the status of cigarette smoking, alcohol consumption, and betel quid chewing into three levels: no, occasional smoking/drinking/chewing, and regular smoking (smoking one or more than one pack per day)/drinking/chewing. Data on physical activity were also defined as three levels: no, moderate, and sufficient activity. Each behavior (smoking, drinking, betel quid chewing, and physical activity) was a mutually exclusive categorical variable.

### MetS and Its Risk Components

The participants underwent a physical examination and routine blood and urine laboratory tests to measure waist circumference, systolic blood pressure, diastolic blood pressure, fasting plasma glucose, high-density lipoprotein cholesterol (HDL-C), and triglycerides. The MetS risk components included central obesity, elevated blood pressure, elevated fasting glucose, elevated fasting triglycerides, and reduced HDL-C [14]. MetS was defined as the presence of 3 or more of its risk components [14]. Table S2 in Multimedia Appendix 1 defines those MetS risk components.

### Statistical Analysis

**Figure 1.**
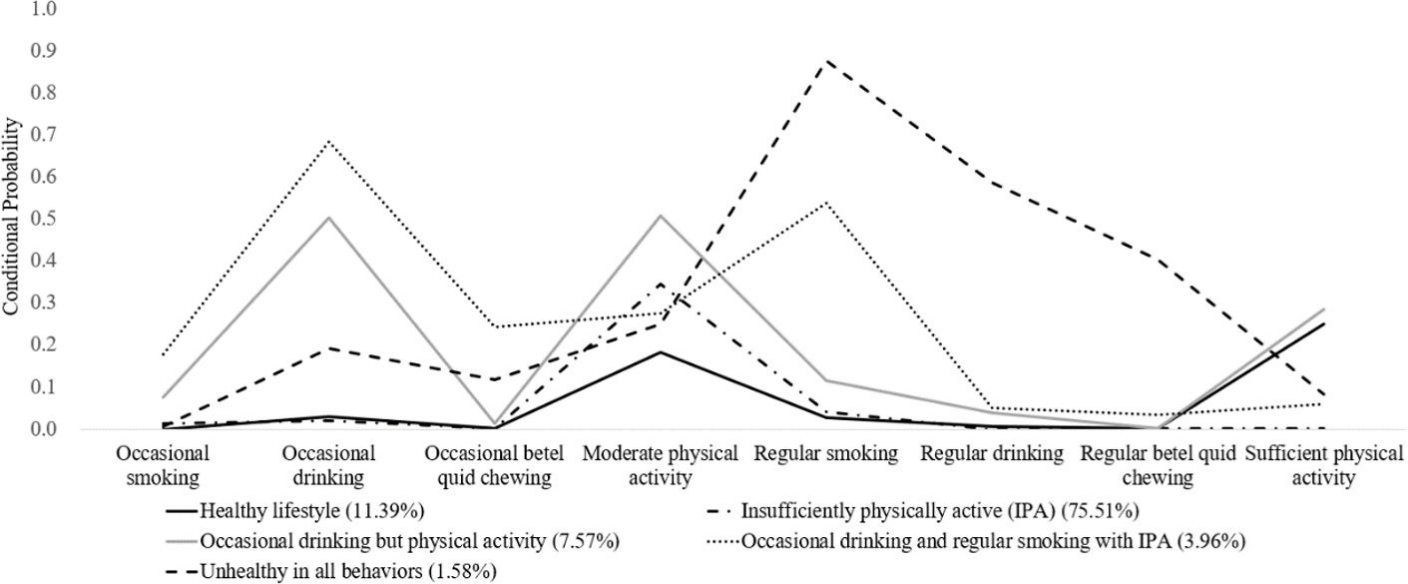
Latent profiles of lifestyle patterns identified by a 5-class model based on latent class analysis using Taiwan’s nationwide claims data from 2020 to 2022.

LCA was performed to categorize patterns of lifestyle. The optimal number of lifestyle patterns was identified by selecting the model with the lowest values of Akaike information criteria, Bayesian information criteria (BIC), consistent Akaike information criterion, and sample-size adjusted BIC, indicating superior model fit [15,16]. The Bayesian factor (BF) is a ratio comparing the likelihood of 2 competing models, where 1<BF<3 indicates weak support, 3<BF<20 indicates moderate support, and BF>20 indicates strong support for the model with K classes compared to a model with a K+1 class [17]. Correct model probability (cmP) estimates the likelihood that each model is the “correct” one among those considered, with the model having the highest cmP being selected [16]. Fit statistics from the 1- to 6-class models to identify appropriate lifestyle patterns are shown in Table S3 in Multimedia Appendix 1. Akaike information criteria, BIC, consistent Akaike information criterion, sample size–adjusted BIC, BF, and cmP values favored the 5-class model. The 5-class model was selected to define patterns of lifestyle in this study. A model with 5 latent classes was chosen: “healthy lifestyle” group, “insufficiently physically active (IPA)” group, “occasional drinking but physically active” group, “occasional drinking and regular smoking with IPA” group, and “unhealthy in all behaviors” group (Figure 1).

This study used the *χ*^2^ test to examine the similarity in the distribution of demographics, lifestyle, and MetS and its risk components between the sampled population and the general population, as well as the differences in demographics and lifestyle across different lifestyle clusters. Binary logistic regression was used to assess the association between lifestyle patterns and MetS risk. We also considered gender and age covariates to adjust in the multivariable regression. The analysis used SAS version 9.4 (SAS Institute Inc). A 2-tailed *P* value of .05 was considered statistically significant.

### Ethical Considerations

This study analyzed secondary administrative data from the APHSD (provided by the Health Promotion Administration, MOHW, Taiwan) and NHI claims data (managed by the HWDC, MOHW, Taiwan). The protocol was approved by the institutional review board of National Cheng Kung University (approval no. 112‐595). All data were fully deidentified and could not be traced to individual identities; thus, informed consent was waived. Analyses were conducted within the secure HWDC environment, with no access to direct identifiers. Only aggregated results were exported, ensuring confidentiality. As no direct contact with participants occurred, no compensation was provided. The manuscript and supplementary materials include no identifiable images, so no image-use consent forms were required.

## Results

Table 1 summarizes the descriptive statistics and bivariate analyses of adults based on LCA. Among the study participants, 57.29% (138,149/241,156) were female, and 46.89% (113,075/241,156) were between 45 and 64 years old. Regarding lifestyle behaviors, 8.34% (20,111/241,156) were regular smokers, 10.57% (25,490/241,156) occasionally consumed alcohol, 2.74% (6604/241,156) occasionally or regularly chewed betel quid, and 57.89% (139,608/241,156) reported no physical activity.

**Table 1.** Demographic and lifestyle profiles of 5 subgroups identified by latent class analysis using Taiwan’s nationwide claims data from 2020 to 2022.

Variables	Total sample (N=241,156)	Healthy lifestyle (n=27,465)	IPA^a^ (n=182,101)	Occasional drinking but physically active (n=18,244)	Occasional drinking and regular smoking with IPA (n=9539)		Unhealthy in all behaviors (n=3807)	*P* value
Gender, n (%)								<.001
Female	138,149 (57.29)	16,429 (59.82)	113,568 (62.37)	5854 (32.09)	1756 (18.41)		542 (14.24)	
Male	103,007 (42.71)	11,036 (40.18)	68,533 (37.63)	12,390 (67.91)	7783 (81.59)		3265 (85.76)	
Age (y), n (%)								<.001
40‐44	24,483 (10.15)	1377 (5.01)	18,416 (10.11)	2356 (12.91)	1684 (17.65)		650 (17.07)	
45‐54	53,087 (22.01)	4197 (15.28)	39,315 (21.59)	4982 (27.31)	3310 (34.70)		1283 (33.70)	
55‐64	59,988 (24.88)	6660 (24.25)	44,398 (24.38)	5115 (28.04)	2690 (28.20)		1125 (29.55)	
65‐74	65,711 (27.25)	10,440 (38.01)	48,810 (26.80)	4363 (23.91)	1502 (15.75)		596 (15.66)	
75‐84	28,442 (11.79)	4071 (14.82)	22,711 (12.47)	1219 (6.68)	308 (3.23)		133 (3.49)	
85+	9445 (3.92)	720 (2.62)	8451 (4.64)	209 (1.15)	45 (0.47)		20 (0.53)	
Lifestyle, n (%)								
Cigarette smoking								<.001
No	215,852 (89.51)	26,410 (96.16)	171,551 (94.21)	16,270 (89.18)	1496 (15.68)		125 (3.28)	
Occasional	5193 (2.15)	0 (0)	2155 (1.18)	1370 (7.51)	1655 (17.35)		13 (0.34)	
Regular	20,111 (8.34)	1055 (3.84)	8395 (4.61)	604 (3.31)	6388 (66.97)		3669 (96.38)	
Alcohol consumption								<.001
No	210,877 (87.44)	267,67 (97.46)	182,101 (100)	314 (1.72)	1282 (13.44)		413 (10.85)	
Occasional	25,490 (10.57)	0 (0)	0 (0)	16,973 (93.03)	8064 (84.54)		453 (11.90)	
Regular	4789 (1.99)	698 (2.54)	0 (0)	957 (5.25)	193 (2.02)		2941 (77.25)	
Betel quid chewing								<.001
No	234,552 (97.26)	27,442 (99.92)	181,922 (99.90)	18,164 (99.56)	5543 (58.11)		1481 (38.90)	
Occasional	4194 (1.74)	0 (0)	0 (0)	68 (0.37)	3763 (39.45)		363 (9.54)	
Regular	2410 (1.00)	23 (0.08)	179 (0.10)	12 (0.07)	233 (2.44)		1963 (51.56)	
Physical activity								<.001
No	139,608 (57.89)	698 (2.54)	124,138 (68.17)	5880 (32.23)	6413 (67.23)		2479 (65.12)	
Occasional	69,285 (28.73)	0 (0)	57,963 (31.83)	7455 (40.86)	2886 (30.25)		981 (25.77)	
Regular	32,263 (13.38)	26,767 (97.46)	0 (0)	4909 (26.91)	240 (2.52)		347 (9.11)	

a IPA: insufficiently physically active.

Each pattern’s demographic and lifestyle characteristics varied significantly (Figure 1; Table 1). The class prevalence of the IPA group was 75.51% (182,101/241,156), indicating suboptimal health among participants who had low likelihood of occasional or regular smoking, consuming alcohol, or chewing betel quid, as well as a low probability of sufficient physical activity. Approximately 11.39% (27,465/241,156) of the study participants, classified as the “healthy lifestyle” group, had a very low likelihood of smoking, alcohol consumption, and betel quid chewing and a higher likelihood of engaging in sufficient physical activity. The “occasional drinking but physically active” group, with the second highest probability of occasional drinking and the highest probabilities of moderate and sufficient physical activity, represented 7.57% (18,244/241,156) of the study population. The “occasional drinking and regular smoking with IPA” group comprised 3.96% (9539/241,156) of the study population. Only a few participants (1.58%, 3807/241,156) were categorized as the “unhealthy in all behaviors” group, characterized by high likelihoods of regular drinking, smoking, betel quid chewing, and insufficient physical activity. Males and middle-aged individuals were significantly overrepresented in the “occasional drinking and regular smoking with IPA” group, as well as the “unhealthy in all behaviors” group. Those in the “occasional drinking and regular smoking with IPA” group and the “unhealthy in all behaviors” group had more unhealthy behaviors in various domains of lifestyle (*P*<.001).

Among the total sample of 241,156 participants, the prevalence of MetS was 35.72% (86,143/241,156) (Table 2). The prevalence of MetS varied across latent classes, being the lowest in the “healthy lifestyle” group at 29.84% (8195/27,465) and the highest in the “unhealthy in all behaviors” group at 47.10% (1793/3807). Central obesity was present in 48.69% (117,425/241,156) of the total sample and was most common in the “unhealthy in all behaviors” group (49.57%, 1887/3807). The “unhealthy in all behaviors” group exhibited the highest prevalence of elevated blood pressure, with 65.85% (2507/3807) affected. Elevated fasting blood glucose was prevalent in 41.17% (99,278/241,156) and was most common in the “unhealthy in all behaviors” group (45.50%, 1732/3807) and in the “occasional drinking and regular smoking with IPA” group (44.17%, 4213/9539). Elevated fasting triglycerides were prevalent in 29.47% (71,069/241,156) of the sample, notably increasing in the “unhealthy in all behaviors” group to 54.40% (2071/3807). Additionally, reduced HDL-C levels were present in 26.40% (63,672/241,156) of the total population, with the highest prevalence seen in the “occasional drinking and regular smoking with IPA” group (30.49%, 2908/9539) and the “IPA” group (27.83%, 50,677/182,101). Overall, MetS and its risk components were significantly higher in the “occasional drinking and regular smoking with IPA” group and the “unhealthy in all behaviors” group.

**Table 2.** Metabolic syndrome and its components in 5 subgroups by latent class analysis using Taiwan’s nationwide claims data from 2020 to 2022.

Variables	Total sample (N=241,156)	Healthy lifestyle (n=27,465)	IPA^a^ (n=182,101)	Occasional drinking but physically active (n=18,244)	Occasional drinking and regular smoking with IPA (n=9539)	Unhealthy in all behaviors (n=3807)	*P* value
Metabolic syndrome, n (%)							<.001
No	155,013 (64.28)	19,270 (70.16)	116,454 (63.95)	12,201 (66.88)	5074 (53.19)	2014 (52.9)	
Yes	86,143 (35.72)	8195 (29.84)	65,647 (36.05)	6043 (33.12)	4465 (46.81)	1793 (47.1)	
Central obesity, n (%)							<.001
No	123,731 (51.31)	15,436 (56.20)	92,093 (50.57)	9695 (53.14)	4587 (48.09)	1920 (50.43)	
Yes	117,425 (48.69)	12,029 (43.80)	90,008 (49.43)	8549 (46.86)	4952 (51.91)	1887 (49.57)	
Elevated blood pressure, n (%)							<.001
No	104,144 (43.19)	11,724 (42.69)	79,800 (43.82)	7698 (42.19)	3622 (37.97)	1300 (34.15)	
Yes	137,012 (56.81)	15,741 (57.31)	102,301 (56.18)	10,546 (57.81)	5917 (62.03)	2507 (65.85)	
Elevated fasting blood glucose, n (%)							<.001
No	141,878 (58.83)	16,521 (60.15)	107,013 (58.77)	10,943 (59.98)	5326 (55.83)	2075 (54.50)	
Yes	99,278 (41.17)	10,944 (39.85)	75,088 (41.23)	7301 (40.02)	4213 (44.17)	1732 (45.50)	
Elevated fasting triglycerides, n (%)							<.001
No	170,087 (70.53)	21,298 (77.55)	129,605 (71.17)	12,675 (69.47)	4773 (50.04)	1736 (45.60)	
Yes	71,069 (29.47)	6167 (22.45)	52,496 (28.83)	5569 (30.53)	4766 (49.96)	2071 (54.40)	
Reduced HDL-C^b^, n (%)							<.001
No	177,484 (73.60)	21,757 (79.22)	131,424 (72.17)	14,915 (81.75)	6631 (69.51)	2757 (72.42)	
Yes	63,672 (26.40)	5708 (20.78)	50,677 (27.83)	3329 (18.25)	2908 (30.49)	1050 (27.58)	

a IPA: insufficiently physically active.

b HDL-C: high-density lipoprotein cholesterol.

MetS and its risk components significantly varied across the groups with different lifestyle patterns (Tables3  4). After adjusting for age and gender, the highest likelihood of MetS and its risk components was observed in participants who regularly smoked, drank alcohol, chewed betel quid, and had insufficient physical activity. Specifically, those in the “unhealthy in all behaviors” group had significantly elevated odds of high fasting triglycerides (adjusted odds ratio [aOR] 3.16, 95% CI 2.95‐3.40; *P*<.001) and MetS overall (adjusted OR 2.38, 95% CI 2.22‐2.55; *P*<.001) compared to the “healthy lifestyle” group. The next highest risk group was those with occasional drinking, regular smoking, and IPA, with an odds ratio of 2.70 (95% CI 2.56‐2.84; *P*<.001) for elevated triglycerides and 2.38 (95% CI 2.26‐2.50; *P*<.001) for MetS. Compared with the “healthy lifestyle” group, the IPA group also consistently displayed elevated risks across all MetS and its risk components, particularly for MetS (aOR 1.41; 95% CI 1.37-1.45; *P*<.001), central obesity (aOR 1.30; 95% CI 1.27-1.34; *P*<.001), elevated triglycerides (aOR 1.40; 95% CI 1.36-1.45; *P*<.001), and reduced HDL-C (aOR 1.46; 95% CI 1.42-1.51; *P*<.001). The “occasional drinking but physically active” group also presents significantly heightened risks for MetS and its components, though with some variation, including a reduced likelihood of low HDL-C (aOR 0.90; 95% CI 0.85-0.94 *P*<.001).

**Table 3.** The likelihood of metabolic syndrome and its risk components (central obesity and elevated blood pressure) between each lifestyle subgroup based on Taiwan’s nationwide claims data from 2020 to 2022.^a^

Variables	Metabolic syndrome, aOR^b^ (95% CI)	Central obesity, aOR (95% CI)	Elevated blood pressure, aOR (95% CI)
Lifestyle subgroup			
Healthy lifestyle	Ref	Ref	Ref
IPA^c^	1.41 (1.37‐1.45)	1.30 (1.27‐1.34)	1.06 (1.04‐1.09)
Occasional drinking but physically active	1.27 (1.21‐1.32)	1.40 (1.34‐1.45)	1.13 (1.09‐1.18)
Occasional drinking and regular smoking with IPA	2.38 (2.26‐2.50)	1.91 (1.82‐2.00)	1.44 (1.37‐1.51)
Unhealthy in all behaviors	2.38 (2.22‐2.55)	1.76 (1.64‐1.89)	1.66 (1.55‐1.79)
Gender			
Female	Ref	Ref	Ref
Male	1.13 (1.11-1.15)	0.66 (0.65-0.67)	1.45 (1.42‐1.47)

a *P*<.001 for all comparisons with the healthy lifestyle group and female group.

b aOR: adjusted odds ratio; age of participants was adjusted in the binary logistic regression model as well.

c IPA: insufficiently physically active.

**Table 4.** The likelihood of metabolic syndrome risk components (elevated fasting blood glucose, elevated fasting triglycerides, and reduced HDL-C^a^) between each lifestyle subgroup based on Taiwan’s nationwide claims data from 2020 to 2022.^b^

Variables	Elevated fasting blood glucose, aOR^c^ (95% CI)	Elevated fasting triglycerides, aOR (95% CI)	Reduced HDL-C, aOR (95% CI)
Lifestyle subgroup			
Healthy lifestyle	Ref	Ref	Ref
IPA^d^	1.19 (1.15‐1.22)	1.40 (1.36‐1.45)	1.46 (1.42‐1.51)
Occasional drinking but physically active	1.12 (1.07‐1.16)	1.28 (1.23‐1.34)	0.90 (0.85‐0.94)
Occasional drinking and regular smoking with IPA	1.43 (1.37‐1.51)	2.70 (2.56‐2.84)	1.81 (1.71‐1.91)
Unhealthy in all behaviors	1.48 (1.38‐1.59)	3.16 (2.95‐3.40)	1.57 (1.46‐1.70)
Gender			
Female	Ref	Ref	Ref
Male	1.42 (1.39‐1.44)	1.58 (1.55‐1.61)	0.91 (0.89‐0.92)

a HDL-C: high-density lipoprotein cholesterol.

b *P*<.001 for all comparisons with the healthy lifestyle group and female group.

c aOR: adjusted odds ratio; age of participants was adjusted in the binary logistic regression model as well.

d IPA: insufficiently physically active.

## Discussion

### Principal Findings

To the best of our knowledge, this research is the first population-based study with a large sample size to examine the associations of MetS and its risk components with latent classes of lifestyle behavior patterns, using a 3-level ordinal measure for each behavior among adults. The findings of this study are generalizable to the adult population in Taiwan. This study identified 5 distinct lifestyle behavior patterns among Taiwanese adults using an LCA approach. Compared to the “healthy lifestyle” group, the IPA group, the “occasional drinking but physically active” group (except for a reduced likelihood of reduced HDL-C), the “occasional drinking and regular smoking with IPA” group, and the “unhealthy in all behaviors” group showed significantly higher odds of developing MetS and its components, with the most potent effects observed in the “occasional drinking and regular smoking with IPA” group and the “unhealthy in all behaviors” group.

Our study showed that the prevalence of MetS was 35.72%, aligning with previous findings of 33% [18]. Chang et al [19] reported a prevalence of 37.9% among individuals over 20 years old in northern Taiwan, while Loke et al [20] observed a prevalence of 34.2% in Taiwanese residents undergoing health examinations at a tertiary medical care facility in southern Taiwan. These results suggest that the prevalence of MetS in Taiwan is slightly higher than in other regions, such as Europe (24.3%) and Japan (23.3% in men, 8.7% in women) [21,22].

Our study revealed clustering patterns of lifestyle behaviors among adults. Notably, approximately 1.58% of participants fell into the “unhealthy in all behaviors” group, characterized by frequent smoking, alcohol consumption, betel quid chewing, and insufficient physical activity. Additionally, 3.96% of participants exhibited occasional drinking and regular smoking, along with insufficient physical activity. These two groups showed the highest odds of MetS and its risk components. Our findings corroborated with previous research, which suggested that the lowest adherence to a healthy lifestyle significantly increased MetS risk [10]. Furthermore, this study presents notable evidence that adopting multiple healthy behavior interventions—including alcohol cessation, smoking cessation, betel nut chewing cessation, and increased physical activity—could be associated with an improved cardiometabolic risk profile and may significantly reduce the risk of MetS. A recent meta-analytic review emphasized the limited impact of single-behavior interventions, which typically yield small-to-medium effect sizes in achieving behavioral or clinical changes, and provided compelling evidence that behavioral change is linearly enhanced as the number of recommendations increases, particularly when the recommendations range between 0 and 4 [12]. Therefore, implementing multibehavior interventions tailored to specific lifestyle behavior patterns is essential for effectively preventing MetS among adults.

Our study found a significant risk of regular smoking on MetS and its risk components, which corroborated with those of previous research. In the Netherlands, Slagter et al [23] demonstrated a positive correlation between smoking and MetS across both genders, irrespective of BMI. Additionally, studies in various demographics have consistently shown that smoking (both current and past smokers) is associated with increased MetS risk [24,25]. The mechanisms linking smoking to MetS are complex. Active smoking disrupts hormonal balance, increases appetite, and gradually reduces metabolic rate [26]. Nicotine exposure is associated with insulin resistance and elevated cortisol, which contributes to abdominal obesity. Smoking also triggers inflammatory responses, as shown by elevated C-reactive protein levels in smokers, a factor linked to type 2 diabetes [27]. Moreover, smoking adversely impacts the lipid profile by increasing total cholesterol and triglycerides and decreasing HDL-C [28]. Notably, a recent meta-analytic review revealed that in the early stages of smoking cessation, the risk of MetS may initially increase compared to active smoking, but it diminishes over time, emphasizing the importance of quitting smoking promptly [29]. Post-cessation obesity may lead to insulin resistance and weight gain, highlighting the need for smoking cessation programs focused on managing weight gain [30].

Moreover, our study identified a link between regular betel quid chewing and increased risks of MetS and its components, consistent with research linking general/central obesity and betel nut chewing [31], which may explain why betel quid chewing was associated with a higher risk of elevated fasting triglycerides and MetS. A longitudinal study by Huang et al [8] using the Taiwan Biobank confirmed that a history of betel nut chewing is associated with MetS and its components, including abdominal obesity and hypertriglyceridemia. This phenomenon may be due to areca alkaloids, such as arecoline, arecaidine, guvacoline, and guvacine, that inhibit the γ-aminobutyric acid receptor, potentially increasing appetite and contributing to obesity [32]. Additionally, betel quid chewing is often accompanied by cigarette smoking and alcohol drinking, and those combinations might also increase the risk of having a cardiometabolic profile.

Notably, a higher likelihood of MetS and its risk components was observed in the IPA group and the “occasional drinking but physically active” group (except for low HDL-C) compared to the “healthy lifestyle” group. Key differences included physical activity levels and occasional drinking prevalence: 97.47% of the “healthy lifestyle” group engaged in sufficient activity, while in the IPA group, none achieved sufficient activity, 31.83% engaged in moderate activity, and 68.17% were inactive. In the “occasional drinking but physically active” group, 26.91% achieved sufficient activity, 40.86% engaged in moderate activity, 32.23% were inactive, and 93.03% reported occasional drinking. Our findings responded to the World Health Organization’s recommendation of at least 150-300 minutes of moderate aerobic activity per week for adults [33]. Physical activity provides protective benefits against MetS. It enhances plasma lipid concentration by raising HDL-C and lowering triglycerides [34,35]. Physical activity also improves glucose tolerance and insulin sensitivity [36], potentially reducing the risk of type 2 diabetes [37]. The IPA group, comprising 75.51% of the study participants, could reduce the likelihood of MetS and its risk components by engaging in sufficient physical activity. Furthermore, our study observed a reduced HDL-C risk in the “occasional drinking but physically active” group. A study in Korea found higher risks of low HDL-C in males who drank alcohol once or less per month but lower risks in females who drank 2‐4 times per month [38]. However, the relationship between occasional drinking and reduced HDL-C remains inconclusive, highlighting the need for further research to explore this association by gender.

### Limitations

This study has some limitations. First, the questionnaire on risk factors underwent validation; however, some measurement errors are inevitable. Second, lifestyle data were obtained from the Adult Preventive Health Services administrative database, which limited the selection of measurement tools; therefore, alternative and potentially more robust instruments could not be employed. Third, sedentary and dietary lifestyles are essential for metabolic health, but they were not accounted for in this study. Additionally, sociodemographic factors, which might influence the likelihood of MetS prevalence, were not considered in this analysis. However, from a health promotion perspective, these factors are generally less modifiable than lifestyle behaviors. Lastly, the study’s cross-sectional design limits the interpretation of results, as it does not establish directionality or causality in the observed relationships.

### Conclusions

This study identified 5 distinct lifestyle behavior patterns, with a MetS prevalence of 35.72%. Compared to the “healthy lifestyle” group, the IPA group, the “occasional drinking but physically active” group, the “occasional drinking and regular smoking with IPA” group, and the “unhealthy in all behaviors” group showed significantly higher odds of developing MetS, with the strongest associations observed in the “occasional drinking and regular smoking with IPA” and “unhealthy in all behaviors” groups. Similarly, all other lifestyle patterns were significantly associated with increased odds of central obesity, elevated blood pressure, elevated fasting blood glucose, elevated fasting triglycerides, and reduced HDL-C relative to the “healthy lifestyle” group. An exception was found for the “occasional drinking but physically active” group, demonstrating a significantly lower HDL-C likelihood. Our results highlight the importance of engaging in sufficient physical activity and implementing multibehavior interventions tailored to specific lifestyle behavior patterns among adults to prevent MetS effectively.

## Supplementary material

10.2196/73114 Multimedia Appendix 1 Supplementary tables with further data on comparisons between the population and the study sample, definitions, and latent cluster analysis.

10.2196/73114 Checklist 1 STROBE checklist for cross-sectional studies.

## Abbreviations

aOR: adjusted odds ratio
APHSD: Adult Preventive Health Services Database
BF: Bayesian factor
BIC: Bayesian information criteria
cmP: correct model probability
HDL-C: high-density lipoprotein cholesterol
HWDC: Health and Welfare Data Science Center
*ICD-10-CM*: *International Classification of Diseases, Tenth Revision, Clinical Modification*
IPA: insufficiently physically active
LCA: latent class analysis
MetS: metabolic syndrome
MOHW: Ministry of Health and Welfare
NHI: National Health Insurance
