# Supplementary material for: Clustering of Lifestyle Behaviors and Their Association With Risk of Metabolic Syndrome Among Adults in Taiwan: Nationwide Cross-Sectional Study
Source: JMIR Public Health Surveill. 2025 Sep 18;11:e73114. doi: 10.2196/73114 (PMC12446567; doi:10.2196/73114)
Supplement: Multimedia Appendix 1 [file publichealth-v11-e73114-s001.docx]

**Table S1.** Comparison of demographics, health behaviors, and metabolic syndrome between the population and the same population.

| Variables | Population | | Sample | | P-Value |
| --- | --- | --- | --- | --- | --- |
|  | N | % | n | % |  |
| Total | 4,019,053 | 100.00 | 241,156 | 100.00 |  |
| Gender |  |  |  |  | 0.999 |
| Female | 2,302,357 | 57.29 | 138,149 | 57.29 |  |
| Male | 1,716,696 | 42.71 | 103,007 | 42.71 |  |
| Age (years) |  |  |  |  | 0.998 |
| 40-44 | 408,041 | 10.15 | 24,483 | 10.15 |  |
| 45-54 | 884,763 | 22.01 | 53,087 | 22.01 |  |
| 55-64 | 999,772 | 24.88 | 59,988 | 24.88 |  |
| 65-74 | 1,095,151 | 27.25 | 65,711 | 27.25 |  |
| 75-84 | 473,985 | 11.79 | 28,442 | 11.79 |  |
| 85+ | 157,341 | 3.91 | 9,445 | 3.92 |  |
| Cigarette smoking |  |  |  |  | 0.594 |
| No | 3,594,860 | 89.45 | 215,852 | 89.51 |  |
| Occasional | 87,444 | 2.17 | 5,193 | 2.15 |  |
| Regular | 336,749 | 8.38 | 20,111 | 8.34 |  |
| Alcohol consumption | |  |  |  | 0.177 |
| No | 3,509,269 | 87.32 | 210,877 | 87.44 |  |
| Occasional | 428,858 | 10.67 | 25,490 | 10.57 |  |
| Regular | 80,926 | 2.01 | 4,789 | 1.99 |  |
| Betel quid chewing |  |  |  |  | 0.686 |
| No | 3,908,079 | 97.24 | 234,552 | 97.26 |  |
| Occasional | 70,857 | 1.76 | 4,194 | 1.74 |  |
| Regular | 40,117 | 1.00 | 2,410 | 1.00 |  |
| Physical activity |  |  |  |  | 0.764 |
| No | 2,324,341 | 57.83 | 139,608 | 57.89 |  |
| Moderate | 1,155,072 | 28.74 | 69,285 | 28.73 |  |
| Sufficient | 539,640 | 13.43 | 32,263 | 13.38 |  |
| Metabolic syndrome | |  |  |  | 0.434 |
| No | 2,582,234 | 64.25 | 155,013 | 64.28 |  |
| Yes | 1,436,819 | 26.86 | 86,143 | 35.72 |  |

**Table S1.** Comparison of demographics, health behaviors, and metabolic syndrome between the population and the same population (cont’)

| Variables | Population | | Sample | | P-Value |
| --- | --- | --- | --- | --- | --- |
|  | N | % | N | % |  |
| Abdominal obesity |  |  |  |  | 0.419 |
| No | 2,058,673 | 51.22 | 123,731 | 51.31 |  |
| Yes | 1,960,380 | 48.78 | 117,425 | 48.69 |  |
| Elevated blood pressure | |  |  |  | 0.722 |
| No | 1,740,038 | 43.29 | 104,144 | 43.19 |  |
| Yes | 2,279,015 | 56.71 | 137,012 | 56.81 |  |
| Elevated fasting blood glucose |  |  |  |  | 0.443 |
| No | 2,361,324 | 58.75 | 141,878 | 58.83 |  |
| Yes | 1,657,729 | 41.25 | 99,278 | 41.17 |  |
| Elevated fasting triglycerides |  |  |  |  | 0.835 |
| No | 2,835,431 | 70.55 | 170,087 | 70.53 |  |
| Yes | 1,183,622 | 29.45 | 71,069 | 29.47 |  |
| Reduced HDL-C |  |  |  |  | 0.917 |
| No | 2,957,522 | 73.59 | 177,484 | 73.60 |  |
| Yes | 1,061,531 | 26.41 | 63,672 | 26.40 |  |

**Table S2.** Definition of metabolic syndrome and its component risks

| **Variable** | **Definition** |
| --- | --- |
| Central obesity | Waist circumference ≥90cm in male or  waist circumference ≥80cm in female |
| Elevated blood pressure | Systolic blood pressure ≥130mmHg or  Diastolic blood pressure ≥85 mmHg |
| Elevated fasting blood glucose | Fasting blood glucose ≥ 100 mg/dL |
| Elevated fasting triglycerides | fasting triglycerides ≥ 150mg/dL |
| Reduced HDL-C ^a^ | HDL<40mg/dL in male or  HDL <50mg/dL in female |
| Metabolic syndrome | Metabolic syndrome is defined as the presence of three or more of the following: central obesity, elevated blood pressure, elevated fasting blood glucose, elevated fasting triglycerides, and reduced HDL |

^a^ HDL-C, High-density lipoprotein cholesterol

**Table S3.** Latent cluster analysis model fit of lifestyle behaviors among participants (n=241,156).

| No. of Classes | df^a^ | LL^b^ | AIC^c^ | BIC^d^ | CAIC^e^ | SABIC^f^ | BF^g^ | cmP^h^ | Proportion of classes |
| --- | --- | --- | --- | --- | --- | --- | --- | --- | --- |
| 1 | 72 | -460387.58 | 49766.98 | 49850.12 | 49858.12 | 49824.70 | 0.00 | 0.00 | 1.00 |
| 2 | 63 | -438345.27 | 5700.36 | 5877.04 | 5894.04 | 5823.02 | 0.00 | 0.00 | 93.88/6.12 |
| 3 | 54 | -436329.47 | 1686.77 | 1957.00 | 1983.00 | 1874.37 | 0.00 | 0.00 | 86.24/9.91/3.85 |
| 4 | 45 | -435691.72 | 429.26 | 793.02 | 828.02 | 681.79 | 0.00 | 0.00 | 86.21/7.54/ 4.68/ 1.58 |
| 5 | 36 | -435581.23 | 226.28 | 683.58 | 727.58 | 543.74 | 149.94 | 1.00 | 75.51/11.39/ 7.57/ 3.96/1.58 |
| 6 | 27 | -435575.56 | 232.94 | 783.78 | 836.78 | 615.35 | 152.04 | 0.00 | 71.25/13.83/5.09/4.37/3.87/1.58 |

^a^ df: degrees of freedom

^b^ LL: log-likelihood

^c^AIC: Akaike information criterion

^d^BIC: Bayesian information criterion

^e^CAIC: consistent Akaike information criterion

^f^SABIC: sample-size adjusted BIC

^g^BF: Bayes factor

^h^cmP: Correct model probability
